# Brain-Derived Neurotrophic Factor (BDNF) in Huntington’s Disease: Neurobiology and Therapeutic Potential

**DOI:** 10.2174/1570159X22666240530105516

**Published:** 2024-05-30

**Authors:** Khairunnuur Fairuz Azman, Rahimah Zakaria

**Affiliations:** 1 Department of Physiology, School of Medical Sciences, Universiti Sains Malaysia, 16150 Kubang Kerian, Kota Bharu, Kelantan, Malaysia

**Keywords:** BDNF, neurotrophin, Huntington’s disease, neurodegeneration, striatum, striatal neurons

## Abstract

Huntington's disease is a hereditary neurodegenerative disorder marked by severe neurodegeneration in the striatum and cortex. Brain-derived neurotrophic factor (BDNF) is a member of the neurotrophin family of growth factors. It plays a crucial role in maintaining the survival and proper function of striatal neurons. Depletion of BDNF has been linked to impairment and death of striatal neurons, leading to the manifestation of motor, cognitive, and behavioral dysfunctions characteristic of Huntington's disease. This review highlights the current update on the neurobiology of BDNF in the pathogenesis of Huntington's disease. The molecular evidence and the affected signaling pathways are also discussed. In addition, the impact of experimental manipulation of BDNF levels and its pharmaceutical potential for Huntington's disease treatment are explicitly reviewed.

## 1 INTRODUCTION

Huntington’s disease (HD) is a hereditary neurodegenerative disease inherited as an autosomal dominant trait caused by the expansion of the CAG repeat in the huntingtin (HTT) gene [1]. When the number of CAG repeats exceeds 36, the translated polyglutamine expansion (polyQ) in the HTT protein (mutant HTT [mHTT]) interferes with the normal functions of major brain cells, including neurons, astrocytes, and microglia [2]. The disease was originally named after George Huntington, who wrote the first detailed description in 1872. Its estimated prevalence in North America, northwestern Europe, and Australia falls between 5.96 to 13.7 cases per 100,000 population, whereby the prevalence in Asia is notably lower compared to Western populations [3]. Moreover, there has been a documented increase in prevalence over the past half-century [4]. The mean proportion of juvenile HD (JHD), defined as HD with an onset of less than 20 years, is less than 5% [5]. Although HD is relatively rare, its impact on patients and their families can be devastating. Treatment options for HD are currently limited, and available data do not indicate any disease-modifying effects for therapeutic interventions in both premanifest and manifest HD populations, while several interventions have demonstrated a clear lack of effectiveness [6]. Similarly, there was no eligible evidence supporting the effectiveness of physiotherapy, occupational therapy, exercise, dietary modifications, or surgical treatments [6]; thus, finding an effective treatment option is imperative.

Neurotrophins are a family of proteins that regulate the function, differentiation, development, and survival of neurons [7]. The classical neurotrophin family comprises structurally similar proteins, including brain-derived neurotrophic factor (BDNF), nerve growth factor (NGF), neurotrophin-3 (NT3), and neurotrophin-4/5 (NT4/5). BDNF is among the most extensively studied and well-characterized neurotrophic factors in the central nervous system. Because of its crucial role in brain development and plasticity, it has become a prominent molecular target for drug development in neurological disorders [8]. Initial evidence implicating BDNF in the pathogenesis of HD dates back to the early 1990s. In an excitotoxic rat model of HD, histological analysis demonstrated the protective effect of BDNF on striatal neurons following intrastriatal injection of adenovirus encoding BDNF [9]. Similarly, grafting cell lines that express BDNF not only prevented the atrophy of all projection neurons but also regulated their phenotype and mitigated degenerative changes in striatal projection neurons following excitotoxicity *in vivo* [10].

The striatum, a key component of the basal ganglia, serves various functions, including the regulation of movement, reward processing, and addiction. BDNF plays a crucial role in determining the size of the striatum by supporting the survival of immature striatal neurons at their origin, promoting the maturation of striatal neurons, and facilitating the establishment of connections within the striatum during brain development [11]. The mutation of HTT results in decreased transcription of the BDNF gene, consequently reducing cortical BDNF production. This leads to insufficient neurotrophic support for striatal neurons, ultimately resulting in their death [12]. The dysfunction and death of striatal neurons serve as the primary causes of the motor disorders associated with HD, including dystonia, chorea, bradykinesia, loss of postural reflexes, and rigidity. This neuronal dysfunction can be restored by modulating BDNF expression. For instance, intrastriatal BDNF infusion has been shown to restore striatal gene expression in BDNF heterozygous mice [13], while systemic delivery of recombinant BDNF in the R6/2 mouse model of HD increased BDNF synthesis, ameliorated neuropathology, and improved neurological function [14]. Therefore, the modulation of BDNF expression and signaling is considered to be another promising candidate for HD treatment. In this review, we present the latest update on the role of BDNF and its downstream signaling pathways in the pathogenesis of HD. The therapeutic potential of BDNF and its modulation for HD treatment are also highlighted.

## 2 NEUROLOGICAL CHANGES IN HUNTINGTON’S DISEASE

The average age of onset of HD is between 35 and 45 years, with a mean disease duration of 16 years [15, 16]. There are several stages of the disease (premanifest, with soft indicators, phenoconversion, and manifest), each of which is marked by a decrease in independence and a greater need for assistance due to a decline in physical and cognitive function as well as the presence of psychiatric symptoms [17]. The common manifestations of HD include motor symptoms (*e.g*., dystonia, chorea, bradykinesia, loss of postural reflexes, rigidity), cognitive impairment (*e.g*., deterioration of executive functions, slowing of thought processing, difficulty with multitasking, deterioration of visuospatial perception, short-term memory), psychiatric symptoms (*e.g*., obsessive-compulsive disorders, depression, irritability, anxiety, apathy), metabolic symptoms (*e.g*., weight loss, sleep disturbance), and other symptoms such as dysphasia and/or dysphagia [1].

These HD manifestations are correlated with neuropathological and structural changes seen in the brains of HD patients. For example, HD motor symptom profiles are linked to neuronal loss in the primary sensory and secondary visual cortices, while neuronal loss across the associational cortices in the frontal, parietal, and temporal lobes is linked to both motor and mood symptom profiles [18]. In HD, the affected areas of the brain include the striatum, entorhinal cortex, neocortex, hippocampal pyramidal neurons, and subiculum [19]. Several imaging and morphometric studies have demonstrated atrophic changes in the neostriatum, white matter, and cerebral cortex in HD [20-23]. The neuropathological changes include cell loss, predominantly in the basal ganglia and neocortex, particularly the cortico-basal ganglia-thalamocortical (CBGTC) loop and the striatal medium-sized spiny neurons (MSNs), also referred to as striatal projection neurons (SPNs) [24, 25]. In addition to MSNs, other types of neurons, such as thalamic neurons and cortical pyramidal neurons (CPNs), are also subject to loss in HD [26, 27]. Various interneuron types are spared in the striatum, except for parvalbumin-expressing fast-spiking interneurons (FSIs) [28, 29]. In post-mortem brain specimens of individuals clinically diagnosed with HD, immunohistochemistry revealed a substantial loss of calbindin 28K-containing neurons in the neostriatum, accompanied by a significant reduction in calbindin D28K immunoreactivity in the substantia nigra [30, 31]. Furthermore, in cases of motor disorders, selective loss of calbindin-D28k interneurons in the human motor cortex was observed, while mood disorders were associated with a loss of calbindin-D28k, calretinin, and parvalbumin interneurons in the anterior cingulate cortex [32]. Recent findings also suggest that dysfunction in the thalamostriatal (TS) system, a major glutamatergic input source, contributes to motor symptoms and striatal neuronal subtype degeneration, as evidenced by premature loss of cholinergic interneurons and accelerated dystonic phenotype [33]. Moreover, in a 3-nitropropionic acid (3-NP) pathogenetic rat model, deficiencies in long-term potentiation (LTP) within cholinergic interneurons and losses of depotentiation in striatal spiny neurons were observed [34].

In addition, synaptic damage has been proposed to play a significant role in the pathophysiology of HD. Studies in HD animal models have shown altered exocytosis of presynaptic vesicles and Ca^2+^ influx [35, 36] due to alterations in N-methyl-D-aspartate (NMDA) receptor function [37]. In early HD, there is a loss of presynaptic terminal integrity, which begins in the striatum in the premanifest phase and subsequently spreads extensively to extrastriatal regions in the early manifest phase, which is associated with motor impairment [38]. Electrophysiological studies conducted on two HD mouse models, YAC128 and BACHD, revealed functional alterations such as an increase in spontaneous excitatory postsynaptic currents, which were more selectively observed in direct pathway MSNs during the early stages of the disease [39]. However, in the late stages of the disease, both direct and indirect pathway MSNs were affected [39]. A recent study demonstrated a reduction in mitochondrial mass in striatal synaptosomes and mitochondrial stress in striatal and cortical synapses in HD mouse models, suggesting that synaptic mitochondrial dysfunction also plays a central role in the early disease progression of HD [40]. Therefore, based on these studies, there is accumulating evidence indicating that synaptic disconnection, particularly along excitatory pathways, is widespread and nearly ubiquitous in HD. This supports a critical involvement of the huntingtin protein in synaptic transmission. This has led to the notion that HD is not solely a neurodegenerative disease but rather a synaptopathy [41].

## 3 NEUROBIOLOGY OF BDNF

**Fig. (1):**
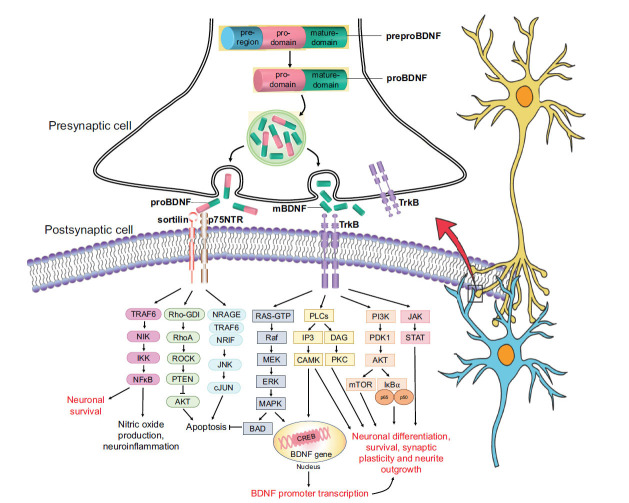
Synaptic BDNF secretion and signaling. BDNF gene produces three functionally different proteins: the precursor preproBDNF, proBDNF, and the mature isoform mBDNF. ProBDNF and mBDNF are released into the extracellular space *via* exocytosis. The mature domain of proBDNF binds to the p75NTR receptor, while the pro-domain binds to the sortilin receptor and activates the TRAF6/NF-kB, PI3K/AKT, and JNK/cJUN signaling pathways. mBDNF binds to the TrkB receptor and activates MAPK, PLCs, PI3K, and JAK/STAT signaling cascades, promoting CREB translation, BDNF promoter transcription, neuronal survival, and synaptic plasticity.

BDNF is widely expressed throughout the brain, including the frontal cortex, cerebellum, thalamus, amygdala, hippocampus, brainstem, basal ganglia, corpus callosum, and pons. The human BDNF gene is located on chromosome 11p13. The BDNF protein is synthesized in the endoplasmic reticulum as a precursor form, preproBDNF [42] (Fig. **1**). After being translocated to the Golgi apparatus, the preproBDNF is subsequently cleaved into the precursor proneurotrophin isoform of BDNF (proBDNF) through signal peptide removal. This protein is composed of 118 amino acids with a mature domain located at the C-terminus and 129 amino acids with an N-terminal pro-domain [43]. The proBDNF protein is further cleaved into its mature form of BDNF by various enzymes. This cleavage process can occur intracellularly through endoproteases from the subtilisin-kexin family, such as furin or convertases [44], or extracellularly *via* plasmin and matrix metalloproteases 2 and 9 [45]. The amino acid sequence of mature human BDNF is identical to that of mouse, rat, and porcine, and it is 90% identical to that of fish, demonstrating that the BDNF gene has been highly conserved throughout the evolution of vertebrates. Depending on the type of cell, the secretion of BDNF into the extracellular space may be activity-dependent or constitutive [43]. ProBDNF and mBDNF are both produced in neuronal cells during cellular membrane depolarization [46]. The phases and areas of brain development dictate the ratio of mBDNF to proBDNF. While mBDNF levels are higher in adulthood, playing a critical role in brain function, such as neuroprotection and synaptic plasticity, proBDNF levels are higher in the early postnatal period, where it is essential for brain function development [47].

The pro-domain of proBDNF binds with the sortilin receptor or other vacuolar protein sorting 10 protein (Vps10p), while the mature domain preferentially binds with the p75 pan-neurotrophin receptor (p75NTR) [48]. The attachment of proBDNF to its specific receptor activates signaling pathways that can control the fate of individual neurons either by promoting its death or survival. The activation of the c-Jun amino-terminal kinase (JNK) pathway by the proBDNF/p75NTR/sortilin binding complex may lead to the loss of the dendritic spine, the release of caspase, and apoptosis of neurons [49]. TNF receptor-associated factor 6 (TRAF6), neurotrophin receptor-interacting factor (NRIF), and neurotrophin receptor-interacting MAGE homolog (NRAGE) are necessary for JNK activation. Additionally, the RhoA/Rho-associated kinase (ROCK) signaling pathway may be activated by proBDNF binding to p75NTR [50]. Activation of ROCK may subsequently activate phosphatase and tension homolog (PTEN), which in turn inhibits phosphoinositide 3-kinase-protein kinase B (PI3K/AKT) signaling required for TrkB-induced potentiation. This cascade of events can lead to apoptosis [51]. The TRAF6 signaling pathway may also be activated by the proBDNF/p75NTR/sortilin binding complex, resulting in the activation of nuclear factor kappa B (NF-kB), which can either promote neuroinflammation or neuronal survival [52].

Conversely, mBDNF binds to the TrkB receptor with a high affinity, causing it to dimerizes. The dimerization of BDNF with the TrkB receptor autophosphorylates intracellular tyrosine residues and activates guanosine triphosphate hydrolases, phospholipase C, PI3K, and Janus kinase signaling pathways [53]. In addition to controlling protein synthesis during neuronal differentiation, the mitogen-activated protein kinase (MAPK)/RAS signaling cascade activates extracellular signal-regulated kinase 1/2 (ERK 1/2) and cAMP response element-binding protein (CREB) [54]. CREB activation leads to initiation of transcription, dendritic arborization, enhancement of long-lasting effects of synaptic potentiation, and neuroprotection [55]. The activation of the PLC signaling pathway by the binding of BDNF to the TrkB receptor results in enhanced synaptic plasticity [56]. This particular pathway is calcium-dependent, whereby it requires the activation of calcium-calmodulin-dependent protein kinase (CAMK) and protein kinase C, causing calcium ions to be released from the intracellular calcium storage. Prosurvival and antiapoptotic effects are mediated by the PI3K/AKT-related pathway, which also affects NMDAR-dependent synaptic plasticity [57]. Moreover, by regulating protein synthesis and cytoskeleton development, the PI3K/Akt/mTOR cascade stimulates dendritic growth and branching [58]. In the major pelvic ganglia, the JAK/STAT pathway stimulates the development of neurite [59]. In general, the binding of BDNF isoforms with various receptor types determines the specialized role of BDNF in regulating brain physiological processes. Perturbation of BDNF synthesis, which can lead to dysfunctions in its signaling cascades, may be implicated in various neurological disorders, including HD.

## 4 NEUROBIOLOGY OF BDNF IN HUNTINGTON’S DISEASE

### 4.1 Animal Studies

Various animal models have been developed and utilized to study the pathogenesis of HD and the association between mHTT and BDNF. Currently, the most popular transgenic rodent models include YAC128, YAC72, R6/1, R6/2, Emx1-Cre/Q140, Emx1-Cre/Q175, N171-82Q, zQ175, HdhQ92, and HdhQ111 mice. In transgenic mouse models of HD overexpressing wild-type (YAC18) or mutant full-length HTT (YAC72), it was observed that full-length wild-type HTT increases the transcription of the BDNF gene. This influences the production and delivery of cortically derived BDNF to striatal targets, whereby the loss of its function in mHTT mice results in decreased production of cortical BDNF, ultimately leading to the death of striatal neurons [12]. Although HTT is widely expressed in the brain, it is highly localized in all cortical pyramidal neurons that project to the striatum as well as striatal neurons projecting to the substantia nigra [60]. Therefore, in HD, the loss of HTT activity resulted in selective vulnerability of these subsets of neurons. Similarly, in Emx1-Cre/Q140 or Emx1-Cre/Q175 heterozygous mouse models, the partial-fusion and full-fusion modes of BDNF-containing vesicles were significantly changed after the onset of HD symptoms, suggesting that abnormal BDNF transcription, transport, and cortical axonal secretion in the striatum contribute to the development of HD [61]. In BDNF^+/−^ and BDNF^−/−^ knockout mice, it has been observed that BDNF is widely distributed in nerve terminals, including brain regions such as the striatum where BDNF messenger RNA is absent, and inhibiting axonal transport or deafferentation depletes BDNF [62]. The decrease in striatal neurons containing parvalbumin corresponds to the reduction in BDNF protein levels, indicating the potential importance of anterograde BDNF transport from neuron cell bodies to terminals for its trafficking in the brain. Additionally, it has been observed that HTT specifically enhances the vesicular transport of BDNF along microtubules [63]. Furthermore, HTT affects the trafficking of BDNF to ligand-bound receptors in striatal neurons, resulting in decreased retrograde transport of TrkB vesicles within striatal dendrites and diminished BDNF/TrkB-induced signaling through c-fos induction and ERK phosphorylation in neurons [64].

Congruently, low BDNF levels were observed in R6/1 and R6/2 mouse models, whereby the deficit of endogenous BDNF modulates the pathology of HD [65, 66]. The decreased levels of BDNF can induce dysfunction in striatal enkephalinergic neurons, leading to severe motor dysfunctions [65]. In the R6/1 mouse model, decreased BDNF expression exacerbates dopaminergic neuronal dysfunction, such as a decrease in retrograde labeling of dopaminergic neurons and striatal dopamine content, resulting in changes in locomotor activity [67]. Decreases in BDNF levels have also been observed in other mouse models, such as N171-82Q and zQ175. In the N171-82Q mouse model of HD, BDNF levels were significantly reduced in brainstem regions containing cardiovascular nuclei [68]. In the zQ175 mouse model, there was a significant decrease in BDNF levels in the striatum, BDNF release in cortical neurons, and the total travel length and speed of BDNF-containing vesicles in neurons [69]. The reduction in BDNF levels is correlated with the disease progression of HD. The blood levels of BDNF protein and messenger RNA (mRNA) were significantly reduced at a symptomatic stage, as seen in an R6/2 mouse model [70]. Similarly, an age-dependent decrease in BDNF mRNA expression occurs in the cerebral cortex and subcortical sources of striatal afferents, including inputs from the midbrain and thalamus [71].

BDNF has been attributed to the pathophysiology of HD *via* several pathways. Previous reports have highlighted functional interactions between BDNF and adenosine A2A receptors (A2ARs), wherein A2ARs promote the excitatory effects of BDNF on hippocampal synaptic transmission [72]. Significant reductions in striatal and hippocampal BDNF were observed in A2AR knockout mice [73]. Moreover, systemic administration of the A2AR antagonist SCH58261 significantly reduced striatal BDNF levels, suggesting that the presence and tonic activation of A2ARs are required for BDNF-induced potentiation of synaptic transmission and maintenance of normal BDNF tone [73]. The ubiquitin-proteasome system (UPS), a cellular process for the non-lysosomal protein degradation of abnormal, oxidized, or misfolded proteins, has also been implicated in HD pathogenesis. Proteasome activities are suppressed in several brain areas and skin fibroblasts of HD patients, whereas enhancing the function of the UPS with a proteasome activator is able to improve cell survival against glutamate toxicity in the HD cell culture model [74, 75]. Furthermore, in R6/2 mice, the excessive CAG repeat lengths are paradoxically associated with elevated proteasome activity, possibly as a cellular compensatory biochemical reaction to the underlying mutation [76].

TrkB signaling dysfunction is another factor contributing to reduced BDNF-mediated trophic support of striatal neurons in HD. Prior to striatal degeneration, the R6/2 mouse model showed early impairments in the levels of the downstream-regulated protein DARPP-32 and activated phospho-TrkB in the striatum [77]. Additionally, BDNF activation of phospho-TrkB and downstream signal transduction was attenuated in R6/2 striatal cultures, suggesting that neurotrophic support of striatal neurons is attenuated early in disease progression due to defects in TrkB signal transduction [77]. The association between proBDNF conversion to mBDNF and the dysregulation of proBDNF receptors, p75NTR, and sortilin with the impairment of striatal neuron survival has also been investigated. There is a significant loss of mBDNF and decreased TrkB activation but no increase in proBDNF or p75NTR levels in either the striatum or the sensorimotor cortex of zQ175 HD mice [78]. However, immature striatal oligodendrocytes have elevated sortilin receptor and p75NTR immunoreactivities, which are linked to substantial myelin abnormalities in the HD striatum. Collectively, this study suggests that, instead of induction in proBDNF, the primary contributing factor to striatal neuron vulnerability in the zQ175 HD mouse model is diminished mBDNF trophic signaling *via* the TrkB receptor.

Furthermore, impaired TrkB receptor signaling has been suggested as one of the mechanisms underlying corticostriatal dysfunction in HD. In early symptomatic HD mouse models, although normal BDNF delivery and TrkB receptor activation occur in the striatum, corticostriatal synaptic dysfunction arises due to a failure of TrkB receptors in movement-suppressing striatal neurons to engage postsynaptic signaling mechanisms [79]. This defect can be corrected by inhibiting p75NTR signaling or its downstream target PTEN, indicating that corticostriatal synaptic dysfunction early in HD is attributable to a correctable defect in the response to BDNF rather than its delivery. The imbalance between p75NTR and TrkB induced by mutant huntingtin in striatal cells, associated with PP1 aberrant activity, disrupts BDNF neuroprotection and likely contributes to the increasing vulnerability of the striatum in HD [80]. Therefore, normalizing TrkB and/or p75NTR signaling or their expression may enhance BDNF neuroprotective therapies in HD.

### 4.2 Human Studies

In combination with the findings from experimental models, the results from studies on HD patients demonstrate a causal relation between BDNF and HD pathogenesis. BDNF gene polymorphisms, such as Val66Met, a methionine (Met) substitution for valine (Val) at codon 66, are associated with alterations in memory and brain anatomy in HD patients. The Val66Met polymorphism modifies the intracellular trafficking and activity-dependent secretion of BDNF in the group of HD patients with 42 and 49 CAG repeats [81]. Moreover, HD patients with a BDNF Val66Met genotype exhibited a later onset compared to those with the BDNF Val66Val genotype [81]. Nevertheless, subsequent studies discovered no association between BDNF gene variants and age at the onset of HD [82-84]. Nevertheless, it is crucial to acknowledge the potential effects of BDNF polymorphism on BDNF transcriptional activity and transport, as well as its impact on the age at onset and progression of HD.

DNA methylation of the BDNF promoter has been the focus of research on indicators of BDNF gene activity in various neurological illnesses. In HD patients, BDNF promoter methylation was increased in blood compared to controls [85]. Downregulation of BDNF promoter II and IV transcription, as well as a significant reduction in BDNF mRNA and protein, were also observed in the human HD cortex from an early symptomatic stage [86]. TrkB mRNA levels are decreased in caudate tissue but remain unchanged in the cortex, whereas the mRNA levels of p75NTR and T-Shc (a truncated TrkB isoform) are elevated in the caudate [86]. This suggests that, in addition to the reduction in BDNF mRNA, there is an imbalance in neurotrophic receptor signaling in HD. Furthermore, differential expression of microRNAs (miRNAs) has been associated with HD. MiRNAs, a class of small noncoding RNAs (sncRNAs), can repress gene expression through translational repression or mRNA deadenylation and decay by base pairing to partially complementary sites. miR-30a-5p and miR-10b-5p were significantly upregulated in HD and targeted the 3’UTR of the BDNF transcript, resulting in the downregulation of BDNF [87]. In conjunction with that, reduced BDNF expression was seen in selected brain regions of HD patients. A reduction of 53 to 82% in BDNF expression was observed in the caudate and putamen, while no reduction was observed in the hippocampus, temporal cortex, or parietal cortex [88]. Additionally, immunohistochemistry analysis revealed decreased BDNF immunoreactivity in caudate neurons, but no such reduction was observed in cortical neurons [88]. These findings suggest selective degradation of BDNF in brain regions susceptible to HD.

Concurrently, reduced BDNF levels were detected in the serum and saliva of HD patients compared to healthy controls [85, 89, 90]. Lower BDNF levels were correlated with longer durations of illness and longer CAG repeat lengths [89]. Nevertheless, inconsistent findings have also been reported. BDNF mRNA and protein levels in the blood or CSF were not significantly different between HD patients and controls [91, 92]. BDNF concentration was also not associated with motor symptoms, cognitive impairment, MRI brain volumetric measures, or clinical scores, and had a poor ability to discriminate controls from HD mutation carriers and premanifest from manifest HD [90, 92]. Moreover, HD patients exhibited moderately increased intraplatelet BDNF levels compared to controls [93]. These varying results urge careful consideration in using peripheral BDNF levels as biomarkers of HD progression; hence, further studies on a larger sample size are warranted to confirm its potential use.

### 4.3 *In vitro* Studies

In sections of the rat cortex and cultured neurons, BDNF mRNA was found to be associated with HTT and components of neuronal RNA granules, which serve as centers for regulating RNA transport and local translation. Normal HTT functions in posttranscriptional repression pathways of BDNF mRNAs through P-bodies or neuronal granules, as well as in its retrograde dynein-mediated transport in dendrites [94]. These findings suggest a role for HTT in neuron survival and neurotrophic support, where impairment of BDNF mRNA sorting and processing is likely involved in the pathogenesis of HD. In HD striatal cells, the cells exhibited decreased release of both pro- and mature BDNF and higher levels of proBDNF [95]. The increased perinuclear labeling of the transduced protein indicates a potential reduction in vesicle processing or transport. This is further supported by reduced postGolgi trafficking of Val-BDNF, but not Met-BDNF, by mHTT [96]. mHTT disrupts BDNF post-Golgi trafficking in the regulated secretory pathway, whereas wild-type HTT promotes trafficking [63]. Furthermore, disruption of axonal transport of BDNF-containing vesicles is observed in cultured hippocampal and striatal neurons but not in cortical neurons [97]. These findings imply that HTT mutation decreases BDNF levels in the striatum by inhibiting gene expression and disrupting the axonal transport of BDNF-containing vesicles to the striatum [98]. A recent study suggests that disrupted spatiotemporal trophic support of BDNF to striatal neurons, caused by impaired transport, could potentially contribute to the pathogenesis of HD [99].

Decreased AKT phosphorylation and increased caspase-3 activation were also observed in HD striatal cells, suggesting the complementary roles of BDNF and TrkB receptors in counteracting the dysfunctional mechanisms underlying HD pathology [95]. Similarly, weight gene correlation network analysis of differentially expressed genes (DEGs) overlapped from HD *versus* control and BDNF-low *versus* high groups, indicating that low BDNF was most strongly correlated with HD [100]. Functional enrichment analyses revealed that DEGs in these modules were significantly enriched in the phagosome, GABAergic synapse, cAMP, MAPK, and Ras signaling pathways [100]. This indicates that diminished BDNF expression and related signaling pathways may play a role in the development of HD.

## 5 MODULATION OF BDNF SIGNALING IN THE TREATMENT OF HUNTINGTON’S DISEASE

Over the years, BDNF has become an essential molecular target in drug development to treat neurodegenerative diseases, including HD. Various approaches have been employed to enhance the clinical outcomes of HD patients by elevating BDNF levels in brain regions crucial for cognition and memory. However, due to its limited diffusion across the blood-brain barrier and short plasma half-life, the use of exogenous delivery of BDNF is limited in clinical practice. To overcome these challenges, a variety of other techniques, including intranasal release of exogenous BDNF, viral gene delivery, and drug-induced increases in endogenous BDNF production, are currently being explored. Other interesting techniques, such as using BDNF mimetic molecules that can effectively stimulate its receptors and pathways, as well as mesenchymal stem cell therapy, seem promising as alternative therapeutic approaches.

### 5.1 Exogenous Administration of BDNF

Although studies using exogenous delivery of BDNF are limited, the intranasal delivery of BDNF has been proven to alleviate anhedonic and depressive symptoms in HD mice. However, BDNF treatment did not increase cell proliferation, neuronal differentiation, or the number of dendritic branches in the hippocampus of the treated animals [101]. Therefore, rather than noninvasive BDNF administration, invasive administration of the BDNF gene is generally a more favorable form of therapy. Table **1** summarizes the exogenous administration of BDNF or BDNF gene delivery. Gene-delivery vehicles are primarily divided into two categories: synthetic carriers (*e.g*., liposomes and polymers) and recombinant viruses (*e.g*., adenovirus, retrovirus, poxvirus, herpes simplex virus, and lentivirus), each with their own advantages and disadvantages. A recombinant virus is the primary means for BDNF gene delivery. Intrastriatal injection of an adeno-associated viral (AAV) vector encoding the BDNF gene increases the production and release of BDNF as well as increases in striatal interneuron support and striatal volume [9, 102-104]. These changes are accompanied by a delay in the onset of the HD mouse motor phenotype and attenuation of both motor and cognitive function impairment [102, 103]. In addition, mice grafted with pGFAP-BDNF astrocytes showed upregulation of BDNF expression and sustained behavioral improvements [105]. The transduction of HD mutant cells with preproBDNF-mCherry (mCh) viral vectors demonstrated the vital role of BDNF-induced TrkB receptor signaling in rescuing HD-mediated apoptotic features in striatal cells through a pathway involving ERK phosphorylation and AKT activation [95].

An alternative strategy for sustained delivery of BDNF involves the utilization of cell-based vectors, such as mesenchymal stem cells (MSCs), neural stem cells (NSCs), fibroblasts, and Schwann cells. MSCs are particularly advantageous due to their reduced immunogenicity, rapid proliferation, and diverse sources, including bone marrow, adipose tissue, umbilical cord, peripheral blood, amniotic fluid, olfactory mucosa, and placenta. The repair mechanisms of MSCs are mainly attributed to antioxidant, immunoregulatory, neurotropic, and antiapoptotic pathways. Interestingly, limited diffusion across the blood-brain barrier can be overcome by utilizing direct BDNF gene delivery using MSCs. A meta-analysis demonstrated a beneficial effect of MSCs on HD rodents overall, evidenced by improvements in muscle strength, motor coordination, morphological changes, cortex-related motor function, neuromuscular electromyography activity, and striatum-related motor function, while cognition was not affected by MSC therapy [106]. In a transgenic mouse model of HD, MSCs engineered to overexpress BDNF exhibited significant therapeutic effects in ameliorating disease progression [107]. Intrastriatal transplantation of MSCs/BDNF decreased anxiety and striatal atrophy while increasing neurogenesis-like activity and the mean lifespan of R6/2 HD mice [108]. The benefits of utilizing MSCs as the delivery platform for BDNF include their ability to secrete a diverse array of neurotrophic and other factors that contribute to the reduction of programmed cell death, inflammation, enhancement of connections between neurons, and mitigation of cell toxicity [109]. In addition, MSCs do not necessitate immunosuppression following allogeneic transplantation and have exhibited a robust and demonstrable safety profile in clinical trials. Intrastriatal grafting of a BDNF-secreting cell line prevented the degeneration of striatal projection neurons, suggesting that a sustained supply of low doses of BDNF could offer therapeutic benefits for the treatment of neurological disorders that impact striatal projection neurons [110]. Similarly, intrastriatal transplantation of embryonic stem cell-derived neural progenitors overexpressing BDNF enhanced neuronal and striatal differentiation, rescued motor function, and preserved adult neurogenesis [111]. Intracerebral transplantation of BDNF-overexpressing human neural stem cells into the contralateral side of unilateral QA-lesioned striatum improved behavior, inflammatory response, and neural networks [112]. However, despite these promising results, the use of cell grafting or viral injection is not feasible for investigating the effect of BDNF on an entire brain region.

Therefore, transgenic overexpression of BDNF in the forebrain has been studied. In this system, a BDNF transgene driven by the promoter for the alpha subunit of Ca^2+^/calmodulin-dependent kinase II (CAMK II) to overexpress BDNF in the forebrain of HD mice was used. In R6/1 HD mice, the BDNF transgene increased striatal TrkB signaling activity and BDNF levels, reversed brain weight loss, ameliorated motor dysfunction, and normalized DARPP-32 expression [113]. In YAC128 HD mice, the BDNF transgene prevented loss and atrophy of striatal neurons and motor dysfunction, improved procedural learning, and normalized expression of the striatal dopamine receptor D2 and enkephalin [114]. In R6/2 HD mice, although apoptosis in the granule cell layer was reduced, elevated BDNF levels were not adequate to restore granule cell survival to its normal levels [115]. In a closely related study utilizing conditional BDNF delivery regulated by the GFAP promoter in astrocytes of R6/2 HD mice, BDNF overexpression prevented a decrease in the levels of striatal BDNF and corticostriatal presynaptic (VGLUT1) and postsynaptic (PSD-95) markers [116]. These changes were correlated with improvements in motor coordination tasks, basal synaptic transmission, synaptic fatigue, and a significant delay in anxiety and clasping alterations [116]. All these results suggest the potential therapeutic value of BDNF overexpression in restoring striatal BDNF levels, thus improving neuronal pathways and HD symptoms. Nevertheless, several concerns about using such therapy should be addressed. For example, several studies have found a link between epileptogenesis and BDNF. Mice overexpressing BDNF throughout the brain exhibited heightened susceptibility to seizures by 16 months of age [114]. This could be explained by the fact that the BDNF transgene utilized in this study is also expressed in epileptogenic regions such as the hippocampus and entorhinal cortex. Consequently, if the BDNF transgene is employed to treat HD patients, it will be vital to avoid expressing it in these brain regions. On the upside, since viral vectors may induce immune responses and inflammation and thus can be considered harmful in clinical trials, gene therapy of the brain is regarded as a relatively safe intervention strategy.

Several *in vitro* studies in brain slices of HD mice have also demonstrated the neuroprotective properties of BDNF in HD pathogenesis. In HdhQ92 and HdhQ111 knock-in mouse hippocampal slices, BDNF restores LTP and synaptic plasticity [117]. In corticostriatal slices of R6/2 mice, BDNF is protected from NMDA-induced toxicity [118]. Similarly, recombinant BDNF improved the levels of BDNF and activated CREB in striatal spiny neurons, leading to improvements in primary outcome measures, including brain volume, size, morphology, atrophy of striatal neurons, microglial reaction, and neuronal intranuclear inclusions [14]. Recent research investigating the effects of exposure to BDNF only or BDNF and NT‐4/5 in cerebral slices discovered that BDNF and NT‐4/5 induce either an antagonistic or synergistic effect on the modulation of corticostriatal synapses, contingent upon the activation of the truncated isoform or the stimulation of the full‐length isoform of TrkB [119].

### 5.2 Stimulation of Endogenous BDNF Production

Transglutaminases (TGases), which catalyze the development of aggregates and cross-link huntingtin, have been proposed to be key players in the etiology of CAG trinucleotide repeat diseases. They make a desirable target for potential therapeutic intervention in HD since TGase activity is elevated in the HD brain. Cystamine is a competitive inhibitor of TGase activity. In the HD mouse model, cystamine has been shown to protect against striatal lesions, volume loss, and neuronal atrophy accompanied by improved behavior and survival [120]. As shown in Table **2**, intraperitoneal injections of 100 mg/kg cystamine or its reduced cysteamine form (a drug approved by the Food and Drug Administration, FDA) into an HD mouse model showed that cystamine increases BDNF secretion from the Golgi region, whereas cysteamine increases BDNF levels in the brain and serum [121]. The argument supporting the use of cystamine and cysteamine as a therapeutic strategy for HD, potentially involving the elevation of BDNF levels, has been reinforced by the assessment of tolerated doses of cystamine in HD patients. Sertraline, a selective serotonin reuptake inhibitor (SSRI), has also been shown to increase brain BDNF levels [122]. Serotonin exerts protective effects on cortical and striatal neurons through the activation of CREB and cyclic AMP signals, which subsequently stimulate BDNF expression. SSRIs function by inhibiting the reuptake of serotonin, thereby enhancing serotonin activity. Accordingly, sertraline has been proven to enhance neurogenesis and ameliorate brain atrophy, thus improving motor performance and prolonging survival [122]. Ampakine, a positive modulator of AMPA-type glutamate receptors, has also been shown to regulate endogenous BDNF levels in HD mice. Twice-daily intraperitoneal injections of a short half-life ampakine normalized BDNF levels and activity-driven actin polymerization in dendritic spines, stabilized LTP, and ameliorated long-term memory impairments [123]. Given that ampakines are well tolerated in clinical trials, these findings point to a fresh approach for the long-term management of the cognitive issues that develop in the early stages of HD.

Small molecule compounds, especially those intended to improve the function of the important physiological pathway, have been repurposed to indirectly increase BDNF levels and, therefore, offer therapeutic potential for HD. CEP-1347, a mixed lineage kinase (MLK) inhibitor, is a semisynthetic compound shown to protect multiple nerve cell types from a variety of insults. Subcutaneous injection of 10 ml/kg CEP-1347 for 4 weeks into R6/2 HD mice increases BDNF levels in the blood through increased transcription of BDNF promoter III [70]. LY-379,268, a potent and selective Group II metabotropic glutamate receptor agonist, restores the motor function phenotype and upregulates BDNF expression in layer 5 neurons within the motor cortex, which project to the striatum, thereby partially mitigating the preferential loss of enkephalinergic striatal neurons and augmenting substance P (SP) expression in SP striatal projection neurons [124]. In a more recent study, subcutaneous injection of 20 mg/kg LY379268 into R6/2 mice protected enkephalinergic striatal projection neurons from degeneration by enhancing BDNF production and delivery through both the thalamostriatal and corticostriatal projection systems [125]. Another compound, N6-cyclohexyladenosine, a selective A1 receptor agonist, has been shown to attenuate neuronal death, neuroinflammation, and oxidative stress and improve cognitive deficits *via* enhanced activation of the PI3K/Akt/CREB/BDNF axis and boost pERK1/2 levels when given intrastriatally at 6.25 nM/1 μL to 3-NP HD rats [126]. Subunits of the cytosolic chaperonin T-complex 1 (TCP-1) ring complex (TRiC or CCT for chaperonin containing TCP-1) have been shown to reduce mHTT levels, rescue defects in BDNF transport and normalize the size of striatal neurons [127]. Moreover, studies on other small molecule compounds that indirectly upregulate BDNF-TrkB signaling are currently emerging. The A2AR agonist inosine upregulates BDNF signaling *via* the TrkB-ERK-CREB pathway and ameliorates motor abnormalities in 3-NP HD rats [128]. The agonist A-971432 activates the sphingosine-1-phosphate receptor 5 (S1PR5) and TrkB downstream AKT and ERK pathways, thus delaying the onset of motor dysfunction in R6/2 mice [129]. Other small molecules indirectly upregulate BDNF expression by exhibiting their action at the TrkB [130, 131], REST [132], and p75NTR [133] pathways, thus offering therapeutic potential in HD.

Currently, several drugs are actively being investigated for the treatment of HD. FK506 (tacrolimus), an immunosuppressant targeting calcineurin function, restored BDNF transport in two complementary models: rat primary neuronal cultures expressing mutant huntingtin and mouse cortical neurons from HdhQ111/Q111 HD knock-in mice [134]. FK506 inhibits calcineurin, the bona fide huntingtin S421 phosphatase, thus restoring BDNF axonal transport defects observed in HD. This finding supports the use of calcineurin as a therapeutic target for HD and offers the first evidence that a drug with FDA approval can restore huntingtin function. Glatiramer acetate, marketed under the brand name Copaxone, among others, is a medication classified as an immunomodulator and is utilized in the treatment of multiple sclerosis. Subcutaneous injection of 250 μg glatiramer acetate into YAC128 and R6/2 HD mouse models increased the expression of functionally active BDNF in astrocytes, decreased neurodegeneration, restored brain BDNF levels, prolonged life span, reduced weight loss, and enhanced motor performance [135]. In another study, subcutaneous injection of glatiramer acetate 0.625 mg/mouse to CAG140 mice or 1 mg/mouse to N171-82Q mice reduced the severity and delayed the onset of HD behavioral symptoms associated with elevated levels of promoter I- and IV-driven BDNF expression and reduced brain cytokines [136].

Vildagliptin (LAF237), an orally active antihyperglycemic agent that selectively inhibits the dipeptidyl peptidase-4 (DPP-4) enzyme, is utilized in the management of type II diabetes mellitus, particularly in cases where GLP-1 secretion and insulinotropic effects are compromised. Oral administration of 5 mg/kg/day vildagliptin to 3-NP HD rats for 14 days significantly improved cognitive and motor perturbations *via* activation of the GLP-1/PI3K/Akt/BDNF pathway [137]. Vildagliptin increased levels of striatal neurotrophic factors and receptors such as BDNF, pS133-CREB, and pY515-TrKB, which subsequently maintained mitochondrial integrity, as evidenced by elevated succinate dehydrogenase (SDH) and cytochrome c oxidase (COX) activities, along with enhancements in the redox modulators Nrf2 and Sirt1 [137]. Roflumilast, commercially known as Daxas among other trade names, is a medication that functions as a selective, long-acting inhibitor of the enzyme phosphodiesterase-4. It is prescribed to reduce the risk of exacerbations in patients with severe chronic obstructive pulmonary disease (COPD) and for the treatment of plaque psoriasis. Oral administration of 0.5, 1, or 2 mg/kg roflumilast to the QA HD rodent model for 21 days significantly improved locomotor activity, attenuated oxidative and nitrosative stress, and decreased elevated proinflammatory cytokines in the striatum and cortex *via* the cAMP/CREB/BDNF signaling pathway [138].

The BDNF-inducing drug Fingolimod (FTY720), an agonist of the sphingosine-1-phosphate receptor, has been demonstrated to modulate dendritic spine density, dendritic architecture, and morphology of healthy mature primary hippocampal neurons in a BDNF-dependent manner [139]. In the R6/2 HD mouse model, chronic administration of Fingolimod prolonged survival, ameliorated motor function, and mitigated brain atrophy while significantly enhancing neuronal connectivity and activity, decreased mutant huntingtin aggregates, and elevated phosphorylation of mutant huntingtin at serine 13/16 residues, predicted to alleviate protein toxicity [140]. Similarly, in the R6/1 HD mouse model, Fingolimod delivery promoted BDNF synthesis, rescued long-term memory deficits, and increased dendritic spine numbers in the hippocampal CA1 area [141]. In the same study, Fingolimod treatment prevented the imbalance of p75NTR/TrkB in the hippocampus through negative modulation of p75NTR, evidenced by increased CREB and TrkB activation and reduced RhoA activity. In another study utilising the R6/1 mouse model of HD, chronic Fingolimod administration from pre-symptomatic stages prevented dendritic spine loss in CA1 hippocampal neurons and improved long-term memory deficits [142]. Furthermore, Fingolimod delivery prevented over-activation of NF-κB signaling and astrogliosis, induced nitric oxide synthase (iNOS) levels, and reduced TNFα, correlated with the normalization of p75NTR expression, thus preventing p75NTR/TrkB imbalance in the hippocampus [142]. Additionally, Fingolimod increased cAMP levels and promoted RhoA and CREB phosphorylation in the hippocampus of R6/1 mice, providing further evidence of its involvement in enhancing synaptic plasticity.

Growing evidence suggests that the prostaglandin E2 (PGE2) EP receptor plays a critical role in the modulation of BDNF and activity-dependent synaptic plasticity. Research has indicated that PGE2 triggers BDNF release in cultured human astrocytes and microglia through EP2 receptor activation [143]. Administration of misoprostol, a PGE2 EP2 receptor agonist, to R6/1 HD mice promotes the expression of hippocampal BDNF, enhances dendritic branching in cultured hippocampal neurons in a BDNF-dependent manner, and alleviates long-term memory deficits [144]. Another receptor agonist that merits investigation is the sigma-1 receptor (S1R) agonist. S1R is located at the endoplasmic reticulum (ER)-mitochondria interface and regulates multiple cellular pathways that are essential for neuronal function that are disrupted in HD. Pridopidine, a selective and potent S1R agonist, is under development by Prilenia Therapeutics and is presently in late-stage clinical trials for HD and amyotrophic lateral sclerosis (ALS). By activating S1R, pridopidine exhibited neuroprotective potential in several models of neurodegenerative disease, including Parkinson's disease, AD, ALS, and HD [145-150]. In particular, it has been demonstrated that pridopidine increases BDNF production in a rat neuroblastoma cell line, increases the expression of genes that are downstream of the BDNF receptor [151], and restores BDNF levels in the brains of R6/2 HD mice [152]. Using a microfluidic device known as a brain-on-a-chip system, pridopidine enhances the availability of corticostriatal BDNF *via* S1R activation, rescues BDNF/TrkB dynamics, and restores synaptic transmission and synapse homeostasis within the HD corticostriatal network, thereby exerting neuroprotective effects [153]. Presently, pridopidine has progressed into HD-focused clinical trials, specifically a multicenter phase III clinical trial that aims to assess the safety and efficacy of pridopidine at a dosage of 45 mg twice daily (BID) in individuals with early-stage manifest HD (NCT04556656).

In recent years, there has been growing interest in the therapeutic potential of naturally occurring compounds due to their proven safety and effectiveness in treating various diseases. Their notable antioxidant capabilities are crucial in combating oxidative stress, which plays a significant role in the pathogenesis of HD. The mutant huntingtin protein directly induces oxidative damage to neurons and astrocytes, contributing to neuronal dysfunction and degeneration [154-156]. Nuclear transcriptional factor-2 (Nrf2) enhancer compounds and Nrf2-regulated vitagenes have been proposed as potential therapeutic agents to treat HD as they activate an endogenous antioxidant pathway that may slow or prevent striatum degeneration [157-159]. Recent studies demonstrated that Nrf2 activation by reversible Kelch-like ECH-associated protein 1 (KEAP1) binding induces the antioxidant response in astrocytes and primary neurons of the zQ175 knockin HD mouse model [160]. Additionally, the administration of diapocynin, an oxidative derivative of the naturally occurring agent apocynin, increased BDNF striatal contents and attenuated 3-NP-induced inflammation, oxidative stress, apoptosis, and gliosis by enhancing the Sirt1/Nrf2 pathway [161].

Furthermore, oral administration of berberine, a naturally occurring compound in goldense, Oregon grape, tree turmeric, and other plants, at 100 mg/kg for 2 weeks to 3-NP HD rats mediates neurotoxicity *via* the activation of BDNF/TrkB/PI3K/Akt signaling attributed to its antioxidant, anti-inflammatory, and anti-apoptotic properties [162]. Morin hydrate is a bioflavonoid mainly obtained from the fruits, stems, and leaves of *Maclura pomifera*, *Maclura tinctoria,* and *Psidium guajava*. Intraperitoneal injection of 10 mg/kg morin hydrate with a combination of calpeptin, a protease inhibitor with selectivity for calpains, into 3-NP HD rats exhibited neuroprotective effects by curbing the Kidins220, glutamate/calpain axis, NF-κB-mediated oxidative stress/neuroinflammation, and the activation of the BDNF/TrkB/Akt/CREB pathway [163]. Tropoflavin, also known as 7,8-dihydroxyflavone, is a naturally occurring flavone present in several sources, such as *Tridax procumbens*, *Godmania aesculifolia*, and primula tree leaves. Oral administration of 5 mg/kg 7,8-dihydroxyflavone to R6/1 mice for 12 weeks delayed motor deficits, reversed memory deficits, improved striatal enkephalin levels, and reduced striatal volume loss *via* selective phosphorylation of the Y816 residue of the BDNF/TrkB receptor in the striatum and activation of the PLCγ1 pathway [164]. Niacinamide or nicotinamide is a water-soluble, active form of vitamin B_3_ (nicotinic acid) found in food (*e.g*., meat, fish, milk, eggs, green vegetables) and used as medication and dietary supplement. Nicotinamide plays essential roles in cell physiology by facilitating NAD+ redox homeostasis and providing NAD+ as a substrate to a class of enzymes that catalyze nonredox reactions. Administration of nicotinamide at 250 mg/kg/day for 12 weeks to the B6.HDR6/1 transgenic mouse model increases the levels of protein BDNF, mRNA BDNF, and peroxisome proliferator-activated receptor gamma coactivator 1-alpha (PGC-1α) and improves motor deficits associated with the HD phenotype [165].

Environmental enrichment, such as changing the surroundings with challenging and stimulating objects, has been demonstrated to significantly delay the progression and onset of HD in transgenic mice [166-169]. Studies on HD patients have also shown these positive effects, whereby environmental enrichment improves physical, mental, and social functioning, as well as self-awareness and self-esteem, even in late-stage HD [170]. The mechanisms underlying these beneficial effects have been linked to the upregulation of neurotrophins such as NGF and BDNF in the cortex and hippocampus. Environmental enrichment, such as voluntary wheel running, larger cages with elevated lids, and various novel objects, in R6/1 HD mice for 8 weeks significantly increased the expression of BDNF in the hippocampus and was independent of the extent of DNA methylation [171]. Another separate study that employed the same model discovered that voluntary wheel running for 10 weeks delayed the decline in cognitive ability and the onset of HD [172]. Although there were no changes in the hippocampal and striatal BDNF protein levels, the BDNF mRNA level in the striatum was improved [172]. These promising effects of voluntary physical exercise and environmental enrichment in the stimulation of endogenous BDNF production imply their therapeutic potential for the treatment of HD.

## CONCLUSION AND FUTURE PROSPECTS

BDNF and its downstream signaling pathways are essential for maintaining the corticostriatal pathway, enhancing synaptic plasticity, delaying the onset, and ameliorating cognitive and motor function impairment in HD. Research indicates that BDNF levels are diminished in individuals with HD, and this decline may play a vital role in the neurodegenerative process characteristic of the disease. Thus, there is a growing interest in the therapeutic potential of BDNF for the treatment of HD. Although the correlation between BDNF and HD is still under investigation, it is evident that reinstating BDNF levels in the striatum or stimulating its downstream signaling pathways could offer therapeutic potential in mitigating the functional impairments experienced by individuals with HD. The preclinical studies on BDNF modulation offer some cause for optimism, albeit cautious, for the therapeutic potential of BDNF in HD. Several approaches are being explored, including the use of drugs that increase BDNF levels, gene therapy to deliver the BDNF gene to the brain, and the use of stem cells that produce BDNF. Primary studies on such therapies in animal models provide compelling evidence that increasing endogenous BDNF production or exogenous BDNF administration may have therapeutic effects. Nevertheless, the application of BDNF therapy needs to be strategized for it to be safe, efficacious, and in an appropriate amount and spatiotemporal context. Furthermore, other alternative therapies, including the consumption of antioxidants derived from natural compounds, environmental enrichment, lifestyle modification, and physical exercise, should be further explored. In addition, future research endeavors should concentrate on comprehending the multifaceted roles of BDNF across different brain regions and conducting closely monitored clinical investigations.

## HIGHLIGHT

This article discussed the neurobiology of brain-derived neurotrophic factor (BDNF) in Huntington's disease. Huntington's disease, characterized by the appearance of defective motor, cognitive, and behavioral traits, arises from a mutation in the huntingtin gene. The resultant production of mutant huntingtin protein subsequently downregulates BDNF expression, leading to dysfunction and death of striatal neurons. This article highlights recent molecular evidence and BDNF signaling pathways implicated in Huntington's disease pathogenesis, as well as the therapeutic potential of experimentally modifying BDNF levels for its treatment.

## Figures and Tables

**Table 1:** Exogenous administration of BDNF for the treatment of Huntington’s disease.

**Therapeutics**	**Mode of Administration, Dose, Duration**	**Study Model**	**Results**	**References**
**BDNF Gene Delivery *via* Viral Vectors and Engineered Cells**				
BDNF-overexpressing human neural stem cells (HB1.F3.BDNF)	Intracerebral transplantation into the contralateral side of unilateral QA-lesioned striatum	QA-lesioned HD rats	HB1.F3.BDNF-treated rats exhibited significant behavioral improvement and reduced inflammatory response. Contralaterally transplanted cells were migrated to the QA-lesioned striatum and differentiated into the cells of GABAergic, MSN-type neurons. Neural networks were established between the transplanted cells and the host brain, and the size of the lateral ventricle was reduced.	[112]
BDNF and neurotrophin-4/5	Cerebral slices exposed to (a) BDNF, (b) BDNF, then NT-4/5, (c) NT-4/5, or (d) NT-4/5 then BDNF for 10 min	3-nitropropionic acid HD mice and COS-7 cell culture	BDNF and neurotrophin-4/5 (NT‐4/5) elicit an antagonistic or synergistic effect on the modulation of corticostriatal synapses that depends on the activation of the truncated isoform or the stimulation of the full‐length isoform of the TrkB	[119]
BDNF-GFP	Embryonic stem cell-derived neural progenitors overexpressing BDNF	Striatum of QA-lesioned, R6/2, and N171-82Q mice	BDNF neural progenitors enhanced neuronal and striatal differentiation, rescued motor function, and preserved adult neurogenesis	[111]
Human MSC/BDNF	Intrastriatal transplantation	R6/2 mice	MSC/BDNF treatment decreased striatal atrophy and anxiety while increasing neurogenesis-like activity and the mean lifespan of the R6/2 mice	[108]
BDNF gene	AAV_1/2_-BDNF, intrastriatal injection	HOMO HD rats	Transfer of the BDNF gene to striatal neurons enhanced BDNF protein levels in the striatum, striatal volume, and NeuN+ cell numbers and attenuated the impairment of both motor and cognitive function.	[102]
BDNF transgene	BDNF transgene driven by the CAMK II alpha subunit promoter	TgBDNF mice, R6/2 mice, and mice carrying both transgenes (R6/2-BDNF)	Although apoptosis in the granule cell layer reduces, increased BDNF was not sufficient to normalize granule cell survival within their normal target in R6/2 mice	[115]
preproBDNF	HD mutant cells transduced with preproBDNF-mCherry (mCh) viral vectors	HD mutant knock-in striatal cells	BDNF-mCherry overexpression rescued decreased Akt phosphorylation, reduced the caspase-3 activation, and enhanced activated ERK in HD cells.	[95]
Recombinant BDNF	4.0 mg per 24 h (152 mg in 100 ml per micropump) in phosphate-buffered saline with 0.1% bovine serum albumin	R6/2 mice	Recombinant BDNF improves the levels of activated CREB and BDNF of the striatal spiny neurons and primary outcome measures such as brain volume, striatal atrophy, size and morphology of striatal neurons, neuronal intranuclear inclusions and microglial reaction. BDNF-treated R6/2 mice survived longer and displayed less severe signs of neurological dysfunction.	[14]
pgfa2BDNF vector	Adenoviral vectors, intrastriatal injection	C6BDNF and C6LacZ cells (for transfection), R6/2 mice (for transduction)	BDNF transgene increases the production and release of BDNF associated with a delay of onset of the motor phenotype of the R6/2 HD transgenic mice	[103]
pGFAP-BDNF transgene	Conditional BDNF delivery regulated by the GFAP promoter in astrocyte	R6/2:pGFAP-BDNF mice	BDNF overexpression prevents the decrease in the levels of striatal BDNF, cortico-striatal presynaptic (VGLUT1), and postsynaptic (PSD-95) markers. These changes were correlated with improvements in motor coordination tasks, basal synaptic transmission, synaptic fatigue, and a significant delay in anxiety and clasping alterations	[116]
BDNF-engineered astrocytes	Retroviral vectors, intrastriatal injection, 3 consecutive days	Primary astrocyte cultures of pGFAP-BDNF mice, Adult Swiss nu-nu mice (for transplantation)	Mice grafted with pGFAP-BDNF astrocytes showed upregulation of BDNF expression and sustained behavioral improvements.	[105]
BDNF transgene	BDNF transgene driven by the CAMK II alpha subunit promoter	Forebrain of YAC128 mice	BDNF overexpression prevented loss and atrophy of striatal neurons and motor dysfunction, normalized expression of the striatal dopamine receptor D2 and enkephalin, and improved procedural learning	[114]
BDNF transgene	BDNF transgene driven by the CAMK II alpha subunit promoter	Forebrain of R6/1 mice	BDNF transgene increased striatal BDNF levels and TrkB signaling activity, ameliorated motor dysfunction, reversed brain weight loss, and normalized DARPP-32 expression	[113]
Human recombinant BDNF	Reperfusion pump system, 2 nM, 1 ml/min 1-2 h before recording	HdhQ92 and HdhQ111 knock-in mice hippocampal slice	BDNF restores long-term potentiation (LTP) and synaptic plasticity	[117]
BDNF gene	AAV-BDNF, intrastriatal injection	QA rodent model of HD	AAV-BDNF provides significant neurotrophic support to striatal interneurons	[104]
Recombinant BDNF	Implantation of stable cell lines secreting high levels of recombinant BDNF, NT-3, or NT-4/5 into rat striatum	QA rodent model of HD	The grafting of a BDNF-secreting cell line prevented the loss of striatal projection neurons. BDNF is the most efficient neurotrophin in promoting the survival of striatal projection neurons	[110]
BDNF gene	AAV-BDNF, intrastriatal injection	QA rodent model of HD	AAV-BDNF provides neuronal protection and significantly reduces the loss of striatal neurons	[9]
**Direct Administration**				
Human recombinant BDNF	5 μl of chitosan solution (0.25%) containing 1.66 μg/kg of BDNF, intranasal, once a day for 15 consecutive days	YAC128 mice	BDNF treatment alleviated anhedonic and depressive-like behaviors in the YAC128 HD mice without altering cell proliferation and neuronal differentiation in the hippocampal dentate gyrus	[101]
**Electrophysiology Experiments**				
BDNF	10 ng/ml	Corticostriatal slices from NMDA-induced toxicity in R6/2 mice, C57BL/6 mice (for patch-clamp experiments)	BDNF protected from NMDA-mediated toxicity in the striatum of R6/2 mice	[118]

**Abbreviations**: AAV: adeno-associated virus, Akt: protein kinase B, CAMK: Ca^2+^/calmodulin-dependent protein kinase, CREB: cAMP response element-binding protein, ERK: extracellular signal-regulated kinase, GABA: gamma-aminobutyric acid, GFP: green fluorescent protein, HD: Huntington’s disease, MSC: mesenchymal stem cells, MSN: medium spiny neurons, NMDA: N-methyl-D-aspartate, NPC: neural progenitors cells, NT: neurotrophin, pGFAP: promoter of the glial fibrillary acidic protein, PSD-95: postsynaptic density protein 95, QA: quinolic acid, TrkB: tropomyosin receptor kinase B, VGLUT1: vesicular glutamate transporter 1.

**Table 2:** Stimulation of endogenous BDNF production for the treatment of Huntington’s disease.

**Therapeutics**	**Mode of Administration, Dose, Duration**	**Study Model**			**Specific Actions on BDNF**	**References**
**Drugs**						
N6-cyclohexylade-nosine	6.25 nM/1 μL, intrastriatal injection	3-NP HD rats			N6-cyclohexyladenosine attenuated neuronal death, neuroinflammation, oxidative stress, and improved cognitive deficits *via* enhanced activation of PI3K/Akt/CREB/BDNF axis as well as boosting pERK1/2 levels	[126]
Inosine	200 mg/kg, intraperitoneal injection, 14 days	3-NP HD rats			Inosine attenuated HD-like symptoms in rats *via* the activation of the A2AR/BDNF/TrKB/ERK/ CREB signaling pathway	[128]
Pridopidine	100 nM, 500 nM, 1 μM, and 10 μM, microfluidic device	Striatal and cortical primary cultures from E15.5 WT and Hdh^CAG140/+^ knock-in mouse embryos generated on a C57/BL6J			Pridopidine enhances the availability of corticostriatal BDNF *via* S1R activation, leading to neuroprotective effects	[153]
Roflumilast	0.5, 1, or 2 mg/kg, orally once daily for 21 days	QA rodent model of HD			Roflumilast significantly improved locomotor activity attenuated oxidative and nitrosative stress, decreased elevated pro-inflammatory cytokines in the striatum and cortex of rat brain through the cAMP/CREB/BDNF signaling pathway	[138]
LY379268	20 mg/kg, subcutaneous injection	R6/2 mice			LY379268 protects enkephalinergic striatal projection neurons from loss by boosting BDNF production and delivery *via* both the corticostriatal and thalamostriatal projection systems	[124]
Vildagliptin	5 mg/kg/day; orally for 14 days	3-NP HD rats			Vildagliptin improved cognitive and motor perturbations in the 3NP rat model *via* activation of the GLP-1/PI3K/Akt pathway	[137]
Glatiramer acetate; Copaxone^®^	CAG140 mice: 0.625 mg/mouse, subcutaneous injection, once a day, 3x per week for 9 months N171-82Q mice: 1 mg/mouse, subcutaneous injection, 5x per week for 12 weeks	CAG140 knock-in and N171-82Q transgenic mice			Glatiramer acetate delayed the onset and reduced the severity of HD behavioral symptoms associated with elevated levels of promoter I- and IV-driven BDNF expression and reduced brain cytokines	[136]
Prostaglandin E2 (PGE2) EP2 receptor agonist; Misoprostol	50 or 500 μg/kg, intraperitoneal injection	R6/1 mice			Misoprostol promotes the expression of hippocampal BDNF, increases dendritic branching in cultured hippocampal neurons in a BDNF-dependent manner, and ameliorates long-term memory deficits	[144]
Glatiramer acetate	250 μg glatiramer acetate in phosphate buffered saline, subcutaneous injection	R6/2 and YAC128 mice			Glatiramer acetate increases the expression of functionally active BDNF in astrocyte culture and in astrocytes of glatiramer acetate-treated HD mice. Glatiramer acetate decreases neurodegeneration, restores brain BDNF levels, reduces weight loss, prolongs life span, and improves motor performance of the treated HD mice	[135]
TRiC subunit	Microfluidic chamber cocultures	E17.5 Primary Embryonic Mouse cortical and striatal neurons			TRiC subunit reduced mHTT, rescued defects in BDNF transport, and normalized the size of striatal neurons	[127]
LY379268	20 mg/kg, subcutaneous injection	R6/2 mice			LY379268 normalizes motor function phenotype and increases BDNF expression in layer 5 neurons in the motor cortex, which project to the striatum, partly rescued a preferential loss of enkephalinergic striatal neurons, and enhanced substance P (SP) expression by SP striatal projection neurons	[125]
FK506	Cells were treated with FK506 (0.1, 0.3, 1 μM) for 30 min before video microscopy	Rat primary neuronal cultures expressing mutant huntingtin and mouse cortical neurons from HdhQ111/Q111 HD knock-in mice			FK506 restored BDNF transport *via* inhibiting calcineurin	[134]
Ampakine	Twice-daily intraperitoneal injections, 5 mg/kg, 8 days	CAG140 mice			Ampakine normalizes BDNF levels, activity-driven actin polymerization in dendritic spines, and LTP stabilization	[123]
CEP-1347	0.5 mg/kg in vehicle; 10 ml/kg subcutaneous injection, 4 weeks	R6/2 mice			CEP-1347, a mixed lineage kinase (MLK) inhibitor, increases BDNF levels in the blood through an increased transcription from BDNF promoter III	[70]
Sertraline	Sertraline hydrocholoride, intraperitoneal injection, 3 days	R6/2 mice			Sertraline prolongs survival, improves motor performance, ameliorates brain atrophy, enhances neurogenesis, and increases brain BDNF levels	[122]
Cystamine, cysteamine	Intraperitoneal injections, 100 mg/kg, gradually increased to reach 300 mg/kg, 1 week.	R6/1, BDNF heterozygous, and double-mutant mice			Cystamine increases BDNF secretion from the Golgi region. Cysteamine increases BDNF levels in the brain and serum	[121]
**Diet/Supplements**						
Berberine	100 mg/kg, orally, 2 weeks	3-NP HD rats			Berberine mediates neurotoxicity *via* its anti-inflammatory, antioxidant, and anti-apoptotic effects, as well as the activation of BDNF/TrkB/PI3K/Akt signaling	[162]
Morin hydrate, calpeptin	Morine hydrate: 10 mg/kg, Calpeptin: 250 μg/kg, intraperitoneal injection, 30 min after the last dose of 3-NP and for 6 days thereafter	3-NP HD rats			Morine hydrate, calpeptin, and their combination exhibited neuroprotective effects against HD through curbing the glutamate/calpain axis, Kidins220, NF-κB-mediated neuroinflammation/oxidative stress, and the activation of BDNF/TrkB/Akt/CREB pathway	[163]
7, 8-dihydroxy-flavone	5mg/kg, orally for 12 weeks	R6/1 mice			7,8-dihydroxyflavone delayed motor deficits reversed memory deficits, improved striatal enkephalin levels, and reduced striatal volume loss *via* selective phosphorylation of Y816 residue of BDNF/TrkB receptor in striatum and activation of PLCγ1 pathway	[164]
Nicotinamide	Mini-osmotic pumps or drinking water deliveries, 250 mg/kg/day for 12 weeks	B6.HDR6/1 transgenic mouse model			Nicotinamide increases the levels of protein BDNF, mRNA BDNF, and peroxisome proliferator-activated receptor gamma coactivator 1-alpha (PGC-1α) and improves motor deficits associated with the HD phenotype	[165]
**Environmental Enrichment**						
Physical exercise and environmental enrichment	Voluntary wheel-running, larger sized cages with elevated lids, various novel objects for 8 weeks	R6/1 mice			Wheel-running significantly increased total BDNF gene expression in the hippocampus. Environmental enrichment significantly increased BDNF expression only in male wild-type animals and was independent of the extent of DNA methylation	[171]
Enhanced physical exercise	Voluntary wheel-running for 10 weeks	R6/1 mice			Voluntary physical exercise delays HD onset and cognitive ability decline. Striatal and hippocampal BDNF protein levels were unchanged. BDNF mRNA level was ameliorated in the striatum	[172]

**Abbreviations**: 3-NP: 3-nitropropionic acid, Akt: protein kinase B, CREB: cAMP response element-binding protein, ERK: extracellular signal-regulated kinase, GLP-1: glucagon-like peptide 1, HD: Huntington’s disease, LTP: long-term potentiation, MLK: mixed lineage kinase, PGC-1α: peroxisome proliferator-activated receptor gamma coactivator 1-alpha, PGE2: prostaglandin E2, PI3K: phosphoinositide 3-kinases, PLCγ1: phospholipase C gamma 1, QA: quinolinic acid, S1R: sigma-1 receptor, SP: substance P, TRiC: chaperonin T-complex 1 (TCP-1) ring complex.

## LIST OF ABBREVIATIONS

3-NP: 3-nitropropionic Acid
A2Ar: Adenosine A2A Receptor
AAV: Adeno-associated Virus
Akt: Protein Kinase B
BDNF: Brain-derived Neurotrophic Factor
CAMK: Ca^2+^/calmodulin-dependent Protein Kinase
CBGTC: Cortico-basal Ganglia-thalamocortical
CREB: cAMP Response Element-binding Protein
ERK: Extracellular Signal-regulated Kinase
FSI: Fast-spiking Interneuron
GABA: Gamma-aminobutyric Acid
GFP: Green Fluorescent Protein
GLP-1: Glucagon-like Peptide 1
HD: Huntington’s Disease
HTT: Huntingtin
JHD: Juvenile HD
JNK: c-Jun Amino-terminal Kinase
LTP: Long-term Potentiation
MAPK: Mitogen-activated Protein Kinase
mBDNF: Mature BDNF
Met: Methionine
mHTT: Mutant HTT
miRNAs: microRNAs
MLK: Mixed Lineage Kinase
mRNA: Messenger RNA
MSC: Mesenchymal Stem Cells
MSN: Medium Spiny Neuron
NF-KB: Nuclear Factor Kappa B
NGF: Nerve Growth Factor
NMDA: N-methyl-D-aspartate
NPC: Neural Progenitors Cell
NRIF: Neurotrophin Receptor-interacting Factor
NSC: Neural Stem Cell
NT: Neurotrophin
NT3: Neurotrophin-3
NT4/5: Neurotrophin-4/5
p75NTR: p75 Pan-neurotrophin Receptor
PGC-1α: Peroxisome Proliferator-activated Receptor Gamma Coactivator 1-alpha
PGE2: Prostaglandin E2
pGFAP: Promoter of the Glial Fibrillary Acidic Protein
PI3K: Phosphoinositide 3-kinases
PLCγ1: Phospholipase C Gamma 1
PSD-95: Postsynaptic Density Protein 95
PTEN: Phosphatase and Tension Homolog
QA: Quinolic Acid
ROCK: RhoA/Rho-associated Kinase
S1R: Sigma-1 Receptor
sncRNA: Small Noncoding RNA
SP: Substance P
SPN: Striatal Projection Neuron
SSRI: Selective Serotonin Reuptake Inhibitor
TG: Transglutaminase
TRAF6: Tumor Necrosis Factor Receptor-associated Factor 6
TRiC: Chaperonin T-complex 1 (TCP-1) Ring Complex
TrkB: Tropomyosin Receptor Kinase B
UPS: Ubiquitin-proteasome System
Val: Valine
VGLUT1: Vesicular Glutamate Transporter 1
Vps10p: Vacuolar Protein Sorting 10 Protein
